# Transcranial temporal interference stimulation precisely targets deep brain regions to regulate eye movements

**DOI:** 10.1007/s12264-025-01387-3

**Published:** 2025-04-11

**Authors:** Mo Wang, Sixian Song, Dan Li, Guangchao Zhao, Yu Luo, Yi Tian, Jiajia Zhang, Quanying Liu, Pengfei Wei

**Affiliations:** 1 Department of Biomedical Engineering, Southern University of Science and Technology, Shenzhen, 518055 China; 2 Brain Cognition and Brain Disease Institute, Shenzhen Institute of Advanced Technology, Chinese Academy of Sciences, Shenzhen, 518055 China; 3 University of Chinese Academy of Sciences, Beijing, 100049 China; 4 Department of Anesthesiology and Perioperative Medicine, Xijing Hospital, Fourth Military Medical University, Xi’an, 710032 China; 5 Shenzhen Zhongkehuayi Technology Co., Ltd, Shenzhen, 518107 China

**Keywords:** Temporal interference stimulation, Superior colliculus, Tissue phantom, Finite element method, Transcranial electrical stimulation, Eye movement

## Abstract

Transcranial temporal interference stimulation (tTIS) is a novel non-invasive neuromodulation technique with the potential to precisely target deep brain structures. This study explores the neural and behavioral effects of tTIS on the superior colliculus (SC), a region involved in eye movement control, in mice. Computational modeling revealed that tTIS delivers more focused stimulation to the SC than traditional transcranial alternating current stimulation. *In vivo* experiments, including Ca^2+^ signal recordings and eye movement tracking, showed that tTIS effectively modulates SC neural activity and induces eye movements. A significant correlation was found between stimulation frequency and saccade frequency, suggesting direct tTIS-induced modulation of SC activity. These results demonstrate the precision of tTIS in targeting deep brain regions and regulating eye movements, highlighting its potential for neuroscientific research and therapeutic applications.

**Supplementary Information:**

The online version contains supplementary material available at 10.1007/s12264-025-01387-3.

## Introduction

Transcranial electrical stimulation (tES) can noninvasively modulate brain regions by injecting weak electrical currents through scalp electrodes, such as the sinusoidal currents in transcranial alternating current stimulation (tACS). Previous studies have increasingly reported that tES of mice can improve cognitive performance in various tasks, including the water maze test [1], the novel object recognition test [2], and the open field test [3]. Evidence from studies in mice supports tES as a potential neuromodulation technique and provides a theoretical foundation for its clinical uses in humans. Recently, tES has been explored as a treatment option for a range of neurological and psychiatric conditions, including depression [4], Parkinson's disease [5], and stroke [6]. Compared to invasive methods such as deep brain stimulation, tES is generally considered safe [7]. However, it has limitations in spatial targeting, particularly for deep brain regions [8–11]. Due to the conductivity of head tissue, the current spreads throughout the brain and decays as it deepens. Therefore, it is challenging for conventional tES to selectively target deep brain regions without activating adjacent areas.

To improve spatial resolution, a new non-invasive electrical stimulation method called transcranial temporal interference stimulation (tTIS) has been proposed [12]. It is based on the hypothesis that neural membranes intrinsically act as a low-pass filter for electrical signal transmission [13]. tTIS applies two high-frequency alternating electrical stimuli at slightly different frequencies (e.g., 2000 Hz and 2001 Hz) to create a low-frequency envelope in specific brain areas [12]. This low-frequency envelope is known as the "beat frequency". Compared to tACS, tTIS can generate more steerable stimulation [14, 15]. It has been reported that tTIS can not only pinpoint specific neuronal populations and small regions of the brain [16] but also subthreshold stimulate deep brain regions such as the hippocampus [17–19], pallidum [20], and thalamus [21]. By stimulating specific neural tissue and assessing the resulting reactions, researchers are able to gain insights into the role of different brain regions. For example, by selectively stimulating the motor cortex, tTIS can induce specific behaviors, such as whisker and forearm movements [16, 22].

Previous studies have reported the robust focality of tTIS [14–16, 22], supporting it as a promising neuromodulatory technique for deep brain regions. The SC, a midbrain structure, is involved in re-orienting vision and maintaining fixation in mammals [23] and is composed of seven or eight layers. The deep layers of mouse SC contain a topographic map of eye movement amplitudes. It has been reported that invasively stimulating the SC with microcurrents can produce eye movements [23], and damage to the SC leads to abnormal saccadic behaviors [24, 25]. However, it is difficult to precisely stimulate the SC region with non-invasive tES methods. To date, there is no evidence that tTIS can effectively activate the SC and modulate saccadic behavior. The causal relationship between tTIS parameters (e.g., intensity and frequency), neural activity, and behaviors (e.g., eye movement) is largely unclear.

In this study, we aimed to investigate the causal effects of tTIS on neural activity and behavior in mice, specifically, neural responses of the SC and eye movements in mice, by recording Ca^2+^ signals in the SC and eye movements concurrently while applying tTIS over the SC. Building upon the foundation of our previous conference abstract about *in vivo* animal experiments [26], we have constructed a phantom model to analyze the impact of different electrode parameters on electrical field distribution. In addition, we have incorporated a finite element simulation model, which, from a modeling perspective, demonstrates that the focality of tTIS is superior under the same parameters as those used in animal experiments. Our hypotheses posit that noninvasive electrical stimulation of the SC can elicit corresponding motor actions in mice through tTIS, and that the parameters of tTIS (e.g., intensity and frequency) can regulate SC neural activity and behavior. To test these hypotheses, we conducted a series of experiments by systematically varying the tTIS parameters and recording their effects on neural activity and eye movements. Our findings indicate that tTIS can effectively activate neural activity in the SC and induce eye movements in mice. Furthermore, we demonstrate that the frequency of tTIS can modulate the frequency of these eye movements.

## Material and methods

### Stimulator and tissue phantom platform

**Fig. 1:**
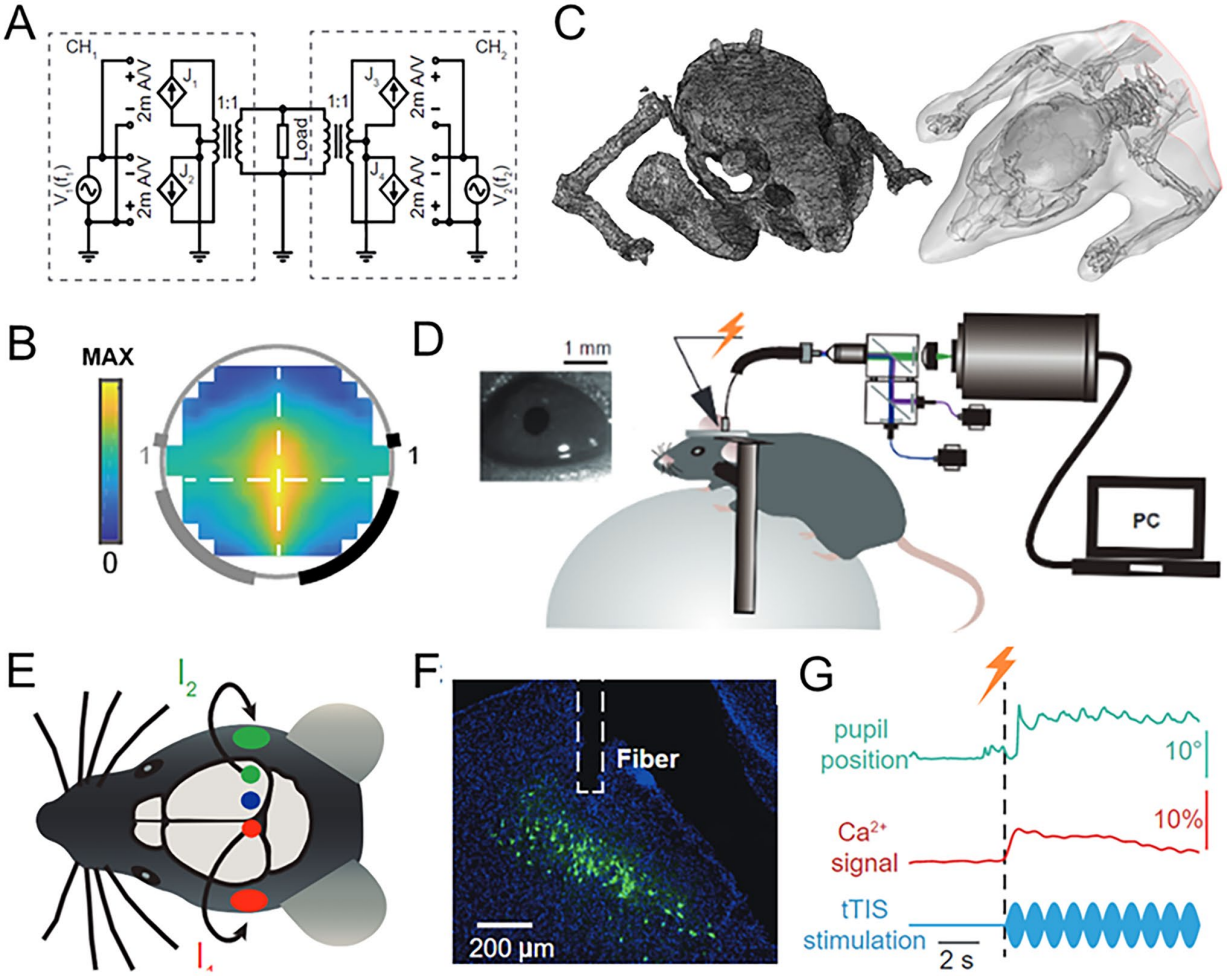
Overview. **A** Design of the tTIS stimulator. **B** Schematic of phantom. **C** Visualization of a finite element model of a mouse brain. **D** Schematic of the experimental setup. tTIS is applied to head-fixed mice. Meanwhile, the value of Ca^2+^ signals in the SC is recorded by fiber photometry, and pupil activity is recorded using a macro lens. **E** Two pairs of tTIS electrodes stimulate a conscious head-fixed mouse with currents I₁ and I_2_. **F** Representative image of GCaMP6s virus expression in the SC of a C57BL/6J mouse (green, GCaMP6s; scale bar, 200 μm). **G** Mean values of eye movement amplitudes (upper green curve) and Ca^2+^ signals in the SC (middle red curve) in three mice during tTIS (carrier frequency: 2000 Hz, difference frequency: 1 Hz, current: 1 mA; lower blue curve).

**Fig. 2:**
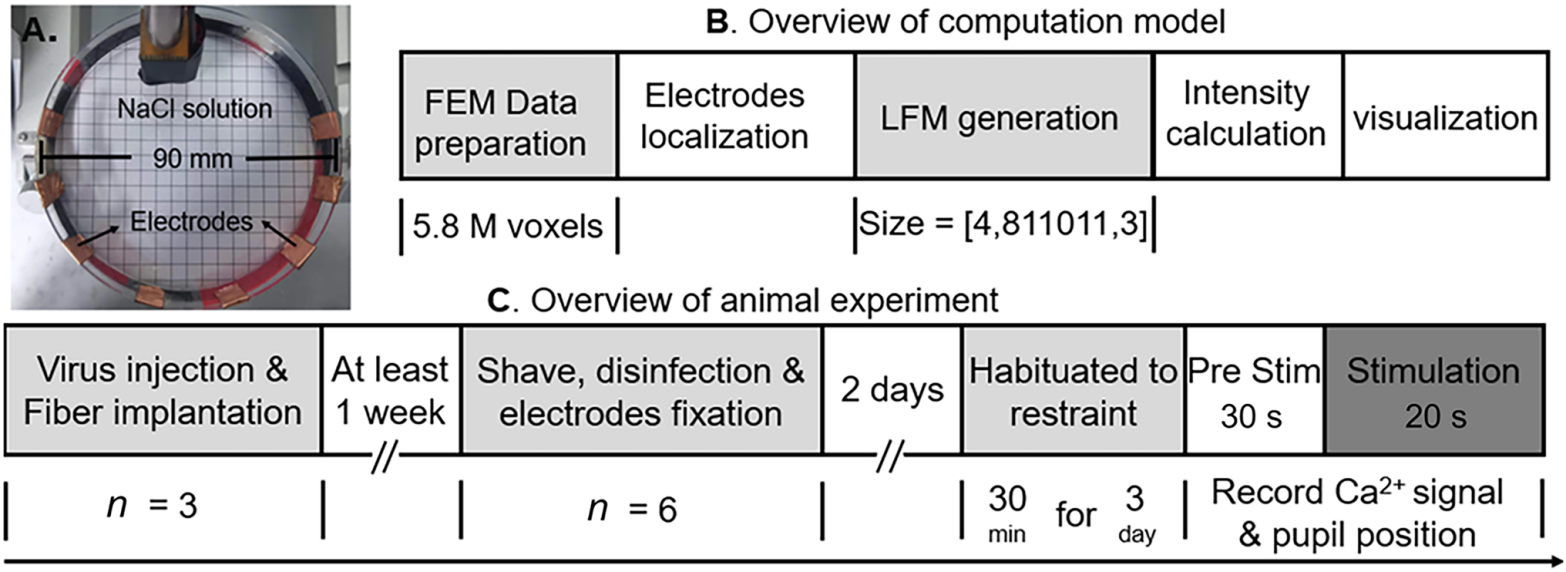
The Experimental Protocol. **A** Experimental Equipment of the Phantom: The tissue model is constructed using a petri dish with a diameter of 90 mm. The tissue model is filled with NaCl solution, and the concentration is adjusted so that the impedance between each pair of copper electrodes is 3 kΩ. **B** Flowchart of the Computational Model: Preparation: An FEM model with 5.8 million voxels is used. The electrode positions are located on the model according to the experimental design, and the Leadfield matrix (LFM) of the brain (811,011 voxels) is calculated. Stimulation: The electrical field strength at the corresponding ROI under different stimulation parameters is calculated. The results are linearly interpolated for better visualization. **C** Flowchart of Animal Experiments: Preparation: Virus injection and fiber optic implantation in half of the mice. One week later, electrode fixation surgery is performed on all mice. Acclimation: After two days, all mice are acclimated for three days (~30 min/day) to get used to the head immobilization system. Data Collection: Ca^2+^ signals and eye movements are recorded 5 s before and 10 s during stimulation.

To evaluate the effects of tTIS, we developed a temporal interference stimulator capable of generating currents with specific frequencies and intensities (Fig. 1A, see supplementary materials for details). In addition, we used a tissue phantom within a cylindrical configuration to mimic the conductivity properties of head tissue (Figs. 1B and 2A).

The tTIS stimulator consists of two independent and controllable constant-current sources, each of which can output a current of 0–5 mA at a frequency of 0–10 kHz with an adjustment accuracy of 0.1 Hz. This source comprises a direct digital synthesis signal generator, an array of constant-current feedback circuits, an inverse reference circuit, and various control circuits (Fig. S1A, B). Such a design ensures a stable current output across a range of loads. The stimulator’s performance underwent extensive testing under different loads, validating its ability to consistently output current and effectively adjust frequencies, even with varying load resistances (Fig. S1C–E).

We then measured the amplitude of interferential electric field envelope modulation using a tissue phantom. This phantom, constructed from a 90 mm diameter dish, features two pairs of copper electrodes symmetrically mounted on the edge of the dish, connected to the tTIS stimulator. Filled with a sodium chloride solution, the salt concentration was adjusted to achieve an interelectrode impedance of 3 kΩ at 2 kHz and 1 mA of alternating current. We also examined the impact of electrode size on the measurements.

The electrical field within the phantom was ascertained using two orthogonal dipole electrodes, spaced 5 mm apart and made from medical stainless-steel needle electrodes. These electrodes were precisely positioned within the phantom using a stereotaxic instrument, advancing in 5-mm steps. The resulting signals were captured on an oscilloscope to record the envelope waveform. To enhance accuracy, measurements at each location were averaged thrice, reducing noise influence. For electrical field mapping, the MatLab interp2 function was applied to linearly interpolate the measurement points, thereby generating comprehensive electrical field maps.

### Simulation-based on the finite element method

We applied the finite element method (FEM) to simulate the electrical fields generated by transcranial electrical stimulation (Fig. 1C). The FEM model was built based on Digimouse [27], a whole-body computed tomography dataset of a mouse with a resolution of 0.1 mm × 0.1 mm × 0.1 mm by Alekseichik *et al.* [28]. To establish the experimental setup, we utilized a pair of copper pins 1 mm in diameter as skull electrodes. Each skull electrode was paired with a 10-mm diameter electrode attached to the ipsilateral cheek. Notably, the needle electrodes did not penetrate the skull, while the cheek electrodes were 5 mm thick saline electrodes on the skin. All electrodes were positioned in the same manner as in the animal experiments. The patch electrode that fitted the cheek was added to the model using SimNIBS 3.2 [29]. Itk-snap [30] and iso2mesh [31] were used to implement the operations of targeting and modifying the FEM model. The model was then converted to segmentation and incorporated the copper pins using Itk-snap [30]. Lastly, the tetrahedral-based FEM model with 5,298,777 tetrahedral elements was loaded using Gmsh [32] and exported as a Nastran Bulk Data File for further processing (Fig. 2B). COMSOL Multiphysics 4.3 (COMSOL, Inc., Burlington, MA) was used to set conductivities and calculate physics equations. Conductivities were defined as follows (in S/m): white matter and grey matter, 0.126; CSF, 1.654; bone, 0.01; scalp, 0.465; eyeballs, 0.5; copper, 6.7; silicone rubber, 29.4; saline, 1.0. The type of study was steady-state current studies and the initial state electric field was set to zero. Based on the model, we generated a lead field matrix (LFM), which served as an intermediary for calculating the field distribution. The LFM enabled the computation of the field generated by any given current injections. Considering that our analysis was limited to the brain area and involved four channels, the resulting matrix had a shape of [4, 811011, 3], where 3 represents the three orthogonal directions.

We performed three simulation experiments, involving the application of 2 mA of both tTIS and tACS. For tACS, the electrical field was directly obtained by injecting current into either two needle electrodes or two pairs of skull-cheek electrodes. On the other hand, to obtain the electrical field distribution for tTIS, we calculated the results for the one-pair skull-cheek electrodes separately. When two high-frequency currents intersect within the brain, they generate interference, resulting in a low-frequency envelope due to the beat frequency phenomenon. Individually, each frequency is too high to drive neural firing effectively [15, 33, 34]. However, this low-frequency beat can modulate neuronal activity. Subsequently, we determined the envelope of the electrical field distribution using the equation (1) as described in [33]:

$$\left|{\overrightarrow{E}}_{AM}\left(\overrightarrow{r}\right)\right|=2\cdot \text{min}\left(\left|{\overrightarrow{E}}_{1}\left(\overrightarrow{r}\right)\right|,\left|{\overrightarrow{E}}_{2}\left(\overrightarrow{r}\right)\right|\right)$$

where $E\left(\overrightarrow{r}\right)$ is the electrical field at the location *r*, and *E*_1_ and *E*_2_ are the electrical fields from one pair of electrodes.

### Animal experiments

#### Experimental preparation

All procedures were approved by the Animal Care and Use Committees of the Shenzhen Institute of Advanced Technology, Chinese Academy of Sciences. An overview of the animal experiments is summarized in Figure 2C. Six adult (4 to 5 months old) male C57BL/6J mice were used. All mice were maintained on a 12/12-h light/dark cycle at 25 °C. Food and water were provided *ad libitum*. The AAV-syn-GCaMP6s virus was used in the fiber photometry experiments. C57BL/6J mice were anesthetized with pentobarbital (1% m/v, 10 mL/kg) and fixed on a stereotaxic apparatus (RWD, Shenzhen, China). During the surgery, the mice were anesthetized with isoflurane (1.5%) and placed on a heating pad to maintain body temperature at 35 °C. A 10 μL microsyringe with a 33-Ga needle was connected to a microliter syringe pump (UMP3/Micro4; WPI, USA) and used for virus injection into the SC (coordinates (in mm): AP, −4; ML, 0.8; DV, −1.6). A 200-μm optical fiber (NA: 0.37; Newdoon, China) was chronically implanted in the SC (coordinates: AP, −4; ML, 0.8; DV, −1.4) of C57BL/6J mice 2–3 weeks following virus expression for fiber photometry experiments. Then, a nylon head plate was implanted into the skull to fix the head. After surgery, all mice were allowed to recover for at least 1 week.

After virus injection and optical fiber implantation, we performed a third operation on C57BL/6J mice (*n =* 3). Moreover, C57BL/6J mice (*n =* 3) without virus injection and optical fiber implantation were subjected to the same procedure. The mice were anesthetized with 4% isoflurane maintained at 1.5%. Erythromycin eye ointment was applied to the eyes. The scalp and face were shaved and disinfected with 70% ethanol. We used two stainless-steel cranial pins (0.8 mm × 4 mm) as electrodes, which were fixed on the skull with dental acrylic. The skull electrodes were located at stereotactic coordinates (relative to bregma) of 4 mm anteroposterior, 2.5 and −1.7 mm mediolateral. Two days later, head-fixed awake mice were habituated to restraint for three consecutive days for 30 min each. During this time, the animals were fixed on top of a polystyrene foam ball. They could remain stationary or run and try to turn the ball forward or backward. According to experimental observations, the mice were largely at rest throughout both the experimental period and the application of electrical stimulation. To prevent mice from getting fatigued, each experiment was completed within 2 h.

#### Histology and microscopy assessments

After the final experiment on each mouse, 1% pentobarbital sodium (10 mL/kg) was injected intraperitoneally for deep anesthesia. Each mouse was transcardially perfused with 1 mol/L phosphate-buffered saline (PBS), followed by ice-cold 4% paraformaldehyde (PFA) in 1 mol/L PBS. The brain was removed and submerged in 4% PFA at 4 °C overnight, post-fixed, and then transferred to 30% sucrose to equilibrate. Coronal brain sections (30 μm) were cut on a cryostat (CM1950; Leica, Germany). The sections were collected and stored in 24 well plates in turn. PBS solution (0.01 mol/L) was added to the plates for short-term storage. The sections of the SC brain area were stained using immunofluorescence as follows: the sections were rinsed with 1 mol/L PBS three times (5 min/time), then incubated with 1:10000 DAPI dye solution for 10 min, and then rinsed with 1 mol/L PBS three times (5 min/time). Next, the sections were attached to the slides, and the anti-fluorescence quenching agent was used to seal the slides. Finally, the Olympus VS120 virtual microscope slide scanning system was used for image observation. The location of the ROI was traced by referring to the mouse whole-brain atlas [35].

#### Neural recording and eye movement recording during tTIS

In this study, Ca^2^⁺ signals were recorded using a fiber photometry system (Inper, Hangzhou, China). Two weeks after the AAV-syn-GCaMP6s virus injection, an optical fiber (NA: 0.37; Newdoon, China) was implanted into the SC. Spontaneous nystagmus refers to those occurring in the absence of any external stimulation. Induced nystagmus, on the other hand, was recorded during the application of electrical stimulation, including changes in gaze direction, either horizontally or vertically (gaze-evoked nystagmus), or from head movements parallel to the plane of the horizontal semicircular canal (head-shaking nystagmus). It can also arise from other types of stimuli, such as vibration (vibration-induced nystagmus), hyperventilation (hyperventilation-induced nystagmus), or changes in posture (positional nystagmus) [36]. To distinguish between the two, we monitored the eye movements during baseline (no stimulation) and compared these to eye movements during electrical stimulation. Spontaneous movements occurred randomly, while induced movements were temporally correlated with the stimulation onset and showed a more consistent directionality and frequency pattern in response to the stimulation. The eye movements of the head-fixed mice were recorded by a 200 Hz macro lens (Point Gray FL3-U3-13EAM) with 1280 × 1024 resolution. The shooting distance was 30 cm, the magnification was 1.6, and the depth of field was >2 mm. We fixed a 940-nm high-power infrared light-emitting diode below the front of the eye for illumination (Fig. 1D). A high transmittance infrared filter was applied to remove the reflection of the infrared lamp on the eyeball for extracting pupil activity.

The head-fixed mice were stimulated using two skull electrodes. During tTIS, two electrically isolated currents I_1_​ and I_2_​ were applied transcranially *via* electrodes connected to the stimulator by thin silver wires (Fig. 1E). Current I_1_​ was applied *via* the skull electrode that was located at the coordinates AP −4 mm, ML 2.5 mm relative to bregma. Current I_2_​ was applied *via* the skull electrode located 4.2 mm laterally to the I_1_​ electrode (distance between centers of electrodes). Each skull electrode was paired with an 8 mm diameter cloth electrode attached to the ipsilateral cheek. The stimulation time was 20 s, repeated five times, with at least 30 s rest time between each trial. During transcranial stimulation, the two cranial electrodes were paired. In the time window of 60 s before and after stimulation, we recorded Ca^2^⁺ signals in the SC with an optical fiber recording system (Inper, Hangzhou, China), and simultaneously photographed the pupil position of mice with a macro lens. We recorded the pupil and Ca^2^⁺ signals of three mice with optical fiber implantation and the pupil data of three mice without optical fiber. A representative image of GCaMP6s virus expression in the SC is shown in Fig. 1F. During the non-stimulation period, Ca^2^⁺ signals and eye movements were minimal, so we used the first 5 s as control data. Upon initial stimulation, both signals and movements increased significantly but stabilized as the stimulation continued. Therefore, for data analysis, we used the first 10 s of stimulation as experimental data. The injected tTIS current, the recorded Ca^2^⁺ signals, and the induced pupil movements are shown in Fig. 1G.

### Data analysis and statistics

First, InperDataProcess V0.2.3 (Inper, Hangzhou) was used to correct the baseline of the original data and reduce the photobleaching caused by long-term recording. We then subtracted the scaled 405-nm trace from the 470 nm trace to generate the corrected 470 nm signal [37]. Custom MatLab (The MathWorks Inc.) scripts were developed for further analysis using R2017a. Signals were analyzed as Δ*F*/*F* = (*F*−*F*_baseline_)/*F*_baseline_, where *F*_baseline_ was defined as the baseline fluorescence within 5 s before stimulation. Then, a Gaussian function was used to smooth the data.

We used the DeepLabCut Toolbox to identify the center of the pupil in the videos [38]. The pupil displacement in the 2D image was converted to a rotation angle based on the estimated eyeball radius (1.67 mm) for adult C57BL/6 mice [39]. We also used a Gaussian function to smooth eye movement data. To analyze the synchronous Ca^2^⁺ signals, all eye movement data were down-sampled to 100 Hz. To quantify eye movement amplitudes, the initial eye position was determined as the average eye position in the 5 s window before tTIS, and the position was set to a 0° angle.

Statistical analyses and graph plotting were performed with Prism 8.0 (GraphPad Software) and MatLab 2017a (MathWorks). Pairwise conditional GC was computed using the multivariate GC MatLab Toolbox [40]. All values are presented as the mean ± SEM. Non-parametric Wilcoxon signed-rank tests were applied for two-group comparisons. *P <0.05, **P <0.01, and ***P <0.001.

## Results

### Effects of TTIS validated by tissue phantom

Our experimental investigations utilizing a tissue phantom filled with sodium chloride solution have delineated the influence of electrode size, position, and current ratio on the electrical field generated by tTIS. The solution’s impedance was calibrated to 3 kΩ at a current frequency of 2 kHz and an amplitude of 1 mA to match the decreased impedance with increasing current frequency [41].

**Fig. 3:**
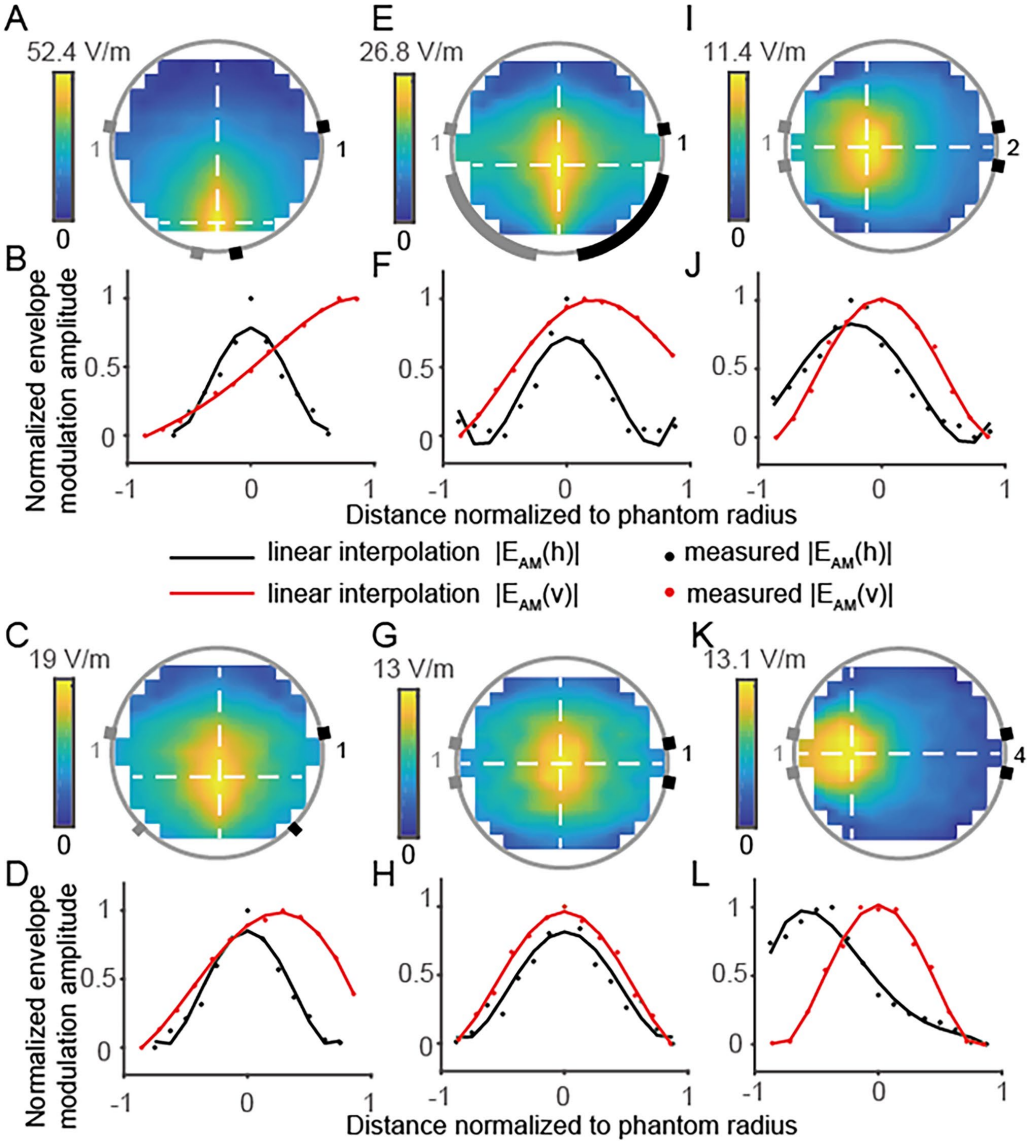
The envelope of the interferential electrical field by tTIS on a cylindrical tissue phantom. **A** Envelope modulation amplitude maps when electrodes are placed in a trapezoidal geometry with a narrow base and the amplitudes of currents I₁ and I_2_ are set to 1 mA. **B** Red and blue lines are horizontal and vertical envelope modulation amplitude along white dotted lines separately, and currents and electrodes are shown in **A**. **C** Envelope modulation amplitude maps when electrodes are placed in a trapezoidal geometry with a wider base and the amplitudes of currents I₁ and I_2_ are set to 1 mA. **D** Envelope modulation amplitude along line cuts. Currents and electrodes are shown in **C**. **E** Envelope modulation amplitude maps with two larger electrodes (10 mm × 50 mm and 10 mm × 50 mm). The amplitudes of currents I₁ and I_2_ are set to 1 mA. **F** Envelope modulation amplitude along line cuts. Currents and electrodes are shown in **E**. **G** Envelope modulation amplitude maps when electrodes are placed in a rectangle with currents I₁ and I_2_ set to 1 mA. **H** Envelope modulation amplitude along line cuts. Currents and electrodes are shown in **G**. **I** Envelope modulation amplitude maps when electrodes are placed in a rectangle with currents in the ratio I₁: I_2_ = 1:2. **J** Envelope modulation amplitude along line cuts. Currents and electrodes are shown in **I**. **K** Envelope modulation amplitude maps when electrodes are placed in a rectangle with currents in the ratio I₁: I_2_ = 1:4. **L** Envelope modulation amplitude along line cuts. Currents and electrodes are shown in **K**.

The interferential electrical field was generated by electrodes positioned as indicated by the rectangular markers in the figures, with the gray and black electrodes forming two separate pairs, carrying alternating currents at frequencies of 2 kHz and 2.001 kHz, respectively. Numbers near the electrodes are current intensities (Fig. 3A, C, E, G, I, K). We further quantified the electrical field envelope amplitude along the white dotted line within the phantom. The envelope modulation amplitudes, normalized to the peak values, are plotted along vertical and horizontal axes in red and black, respectively, with distances normalized to the phantom’s radius and referenced to its center (Fig. 3B, D, F, H, J, L). We report the distribution of the electrical field envelope amplitudes within the phantom, manipulated by varying the electrode placements (Fig. 3A–D), the tTIS current ratio (Fig. 3G–L), and the size of the electrode (Fig. 3C–E). From the results, we learn that the distributions and amplitudes of the electrical field are changed by the changes in both electrode position and the current ratio, which demonstrates that different targets can be stimulated by a set of immobile electrodes. The size of the copper electrodes was 10 mm × 10 mm and 10 mm × 10 mm (Fig. 3A, C, G, I, K), 10 mm × 10 mm, and 10 mm × 50 mm (Fig. 3E). We found that alterations in electrode size did not significantly affect the distribution of electrical field when the electrode positions and current ratios remained constant.

### The tTIS-induced electrical field over the SC region: evidence from the FEM model

**Fig. 4:**
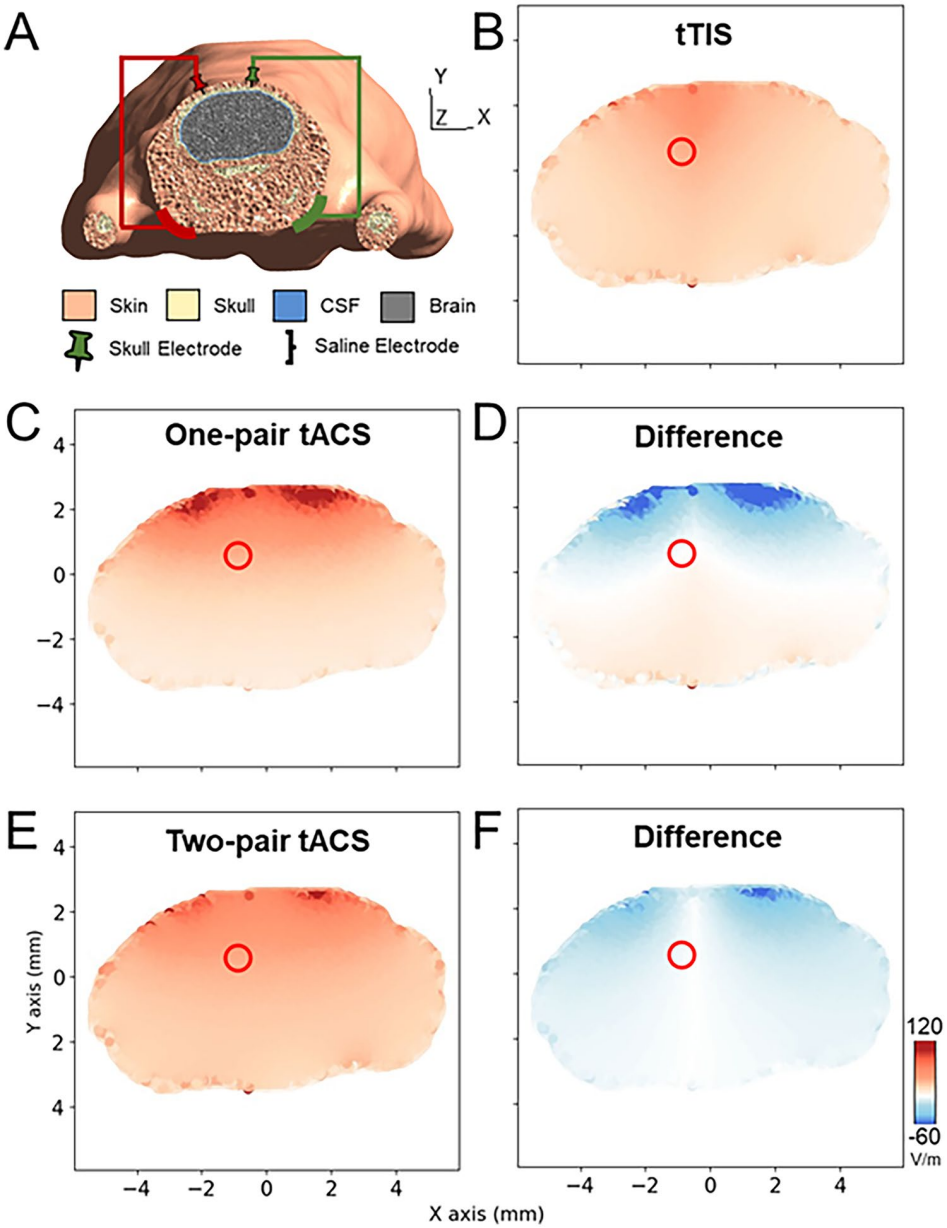
Simulation results**. A** Schematic of Digimouse FEM model. The skull needle electrodes are securely attached to the skull without penetrating it, while patch electrodes are positioned on the skin of the cheeks. **B** An axial view of the electrical field under tTIS. This electrical field is generated by applying high-frequency current (2 mA) through two pairs of electrodes (red and green). **C** The electrical field generated by low-frequency current (2 mA) applied through a single pair of skull electrodes (shaped like needles). **D** The difference in the electrical field between tTIS and one-pair tACS. **E** The electrical field generated by low-frequency current (2 mA) applied through two pairs of electrodes (red and green). **F** The difference in the electrical field between tTIS and two-pair tACS. In all cases, the current across each pair of electrodes is controlled at 1 mA. The red circle indicates the SC region.

For the computational simulation experiment, Fig. 4A displays the FEM model of Digimouse, including the electrode configuration. The resulting electrical fields in the mouse brain, elicited by 2 mA of tTIS/tACS through skull and cloth electrodes, and 2 mA of tACS *via* skull electrodes, are illustrated in Fig. 4B, E, and C, respectively. These simulation results elucidate that placing electrodes solely at the top of the skull results in the convergence of the electrical field within superficial regions, as shown in Fig. 4C and D. Furthermore, the findings highlight that tTIS demonstrates improved effectiveness in achieving a more precise stimulation distribution when applied with identical current intensity and the same electrode configuration as tACS. Notably, this is achieved without compromising the intensity at the desired target, as evidenced in Fig. 4F. These results support the hypothesis that tTIS is more effective in precisely modulating subcortical neural activity.

### Experimental results in awake mice

**Fig. 5:**
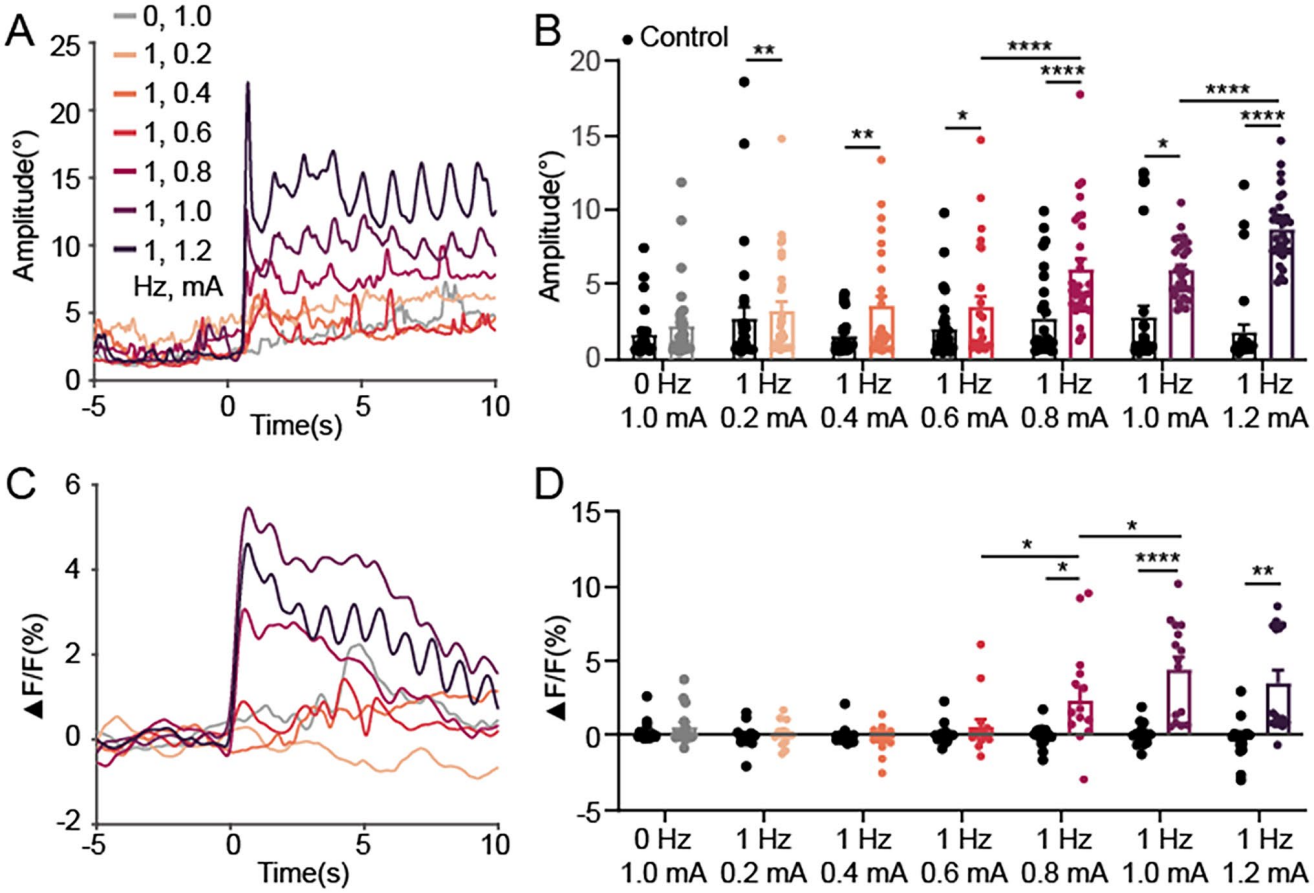
Eye movements and neural activity in the SC respond to tTIS with varying intensities. **A** Eye movement amplitudes respond to tTIS (beat frequency: 1 Hz, carrier frequency: 2000 Hz) with varying intensities (0.2–1.2 mA). Gray line, the control group (beat frequency: 0 Hz, carrier frequency: 2000 Hz, intensity: 1 mA). Sample size: *n =* 6 mice across 30 trials. **B** Comparison of eye movement amplitudes before and during tTIS (control group: average values of eye movement amplitudes within a 3-s window before stimulation; tTIS group: average values of eye movement amplitudes within a 3-s window at the onset of stimulation). Each data point is from an individual trial. *n =* 6 mice across 30 trials. **C** Ca^2+^ signals of neuronal populations in the SC respond to tTIS (beat frequency: 1 Hz, carrier frequency: 2000 Hz) with varying intensities (0.2–1.2 mA). Gray line, the control group (beat frequency: 0 Hz, carrier frequency: 2000 Hz, intensity: 1 mA). *n =* 3 mice across 15 trials. **D** Comparison of Ca^2^⁺ signals in the SC before and during tTIS (control group: average values of Ca^2+^ signal amplitudes in 3-s window before stimulation; tTIS group: average values of Ca^2+^ signal amplitudes in 3-s window at the beginning of stimulation). Each data point is from an individual trial. *n =* 3 mice, 15 trials.

An optical fiber was implanted into the SC of head-fixed mice, and the eye movements were monitored using a macro lens. The exact positioning of the fiber was confirmed by the examination of brain slices from each mouse. The parameters of the fiber and stimulator are presented in Sect. 2.3.3 and Fig. 1E and F. We first investigated whether two high-frequency currents of the same frequency can produce a stimulatory effect in the absence of forming an envelope. Our results showed that tTIS with a current intensity of 1 mA (I_1_: 2000 Hz, 1 mA; I_2_: 2000 Hz, 1 mA) failed to induce eye movements (*n =* 6, 30 trials), neither did it activate neural responses in the SC (*n =* 3, 15 trials) when the beat frequency was 0 Hz. There was no significant difference from the control group (Fig. 5A–D). When applying tTIS with a 1-Hz beat frequency (I₁: 2001 Hz, 1 mA; I_2_: 2000 Hz, 1 mA), eye movements were elicited, and the Ca^2^⁺ signals were increased. They showed a significant difference from the control group (Fig. 5A–D). These results demonstrated that high-frequency currents need an envelope field to stimulate effectively.

We examined the effect of modulating the intensity of tTIS on eye movements and neural activity in the SC. We varied the intensity of tTIS and measured the amplitude of eye movements and Ca^2+^ signals in the SC during tTIS. Our results showed that tTIS with a current intensity ranging from 0.2 mA to 1.2 mA significantly enhanced eye movement amplitudes when the frequency difference was 1 Hz. Only when the current intensity exceeded 0.8 mA were the Ca^2^⁺ signals significantly increased.

**Fig. 6:**
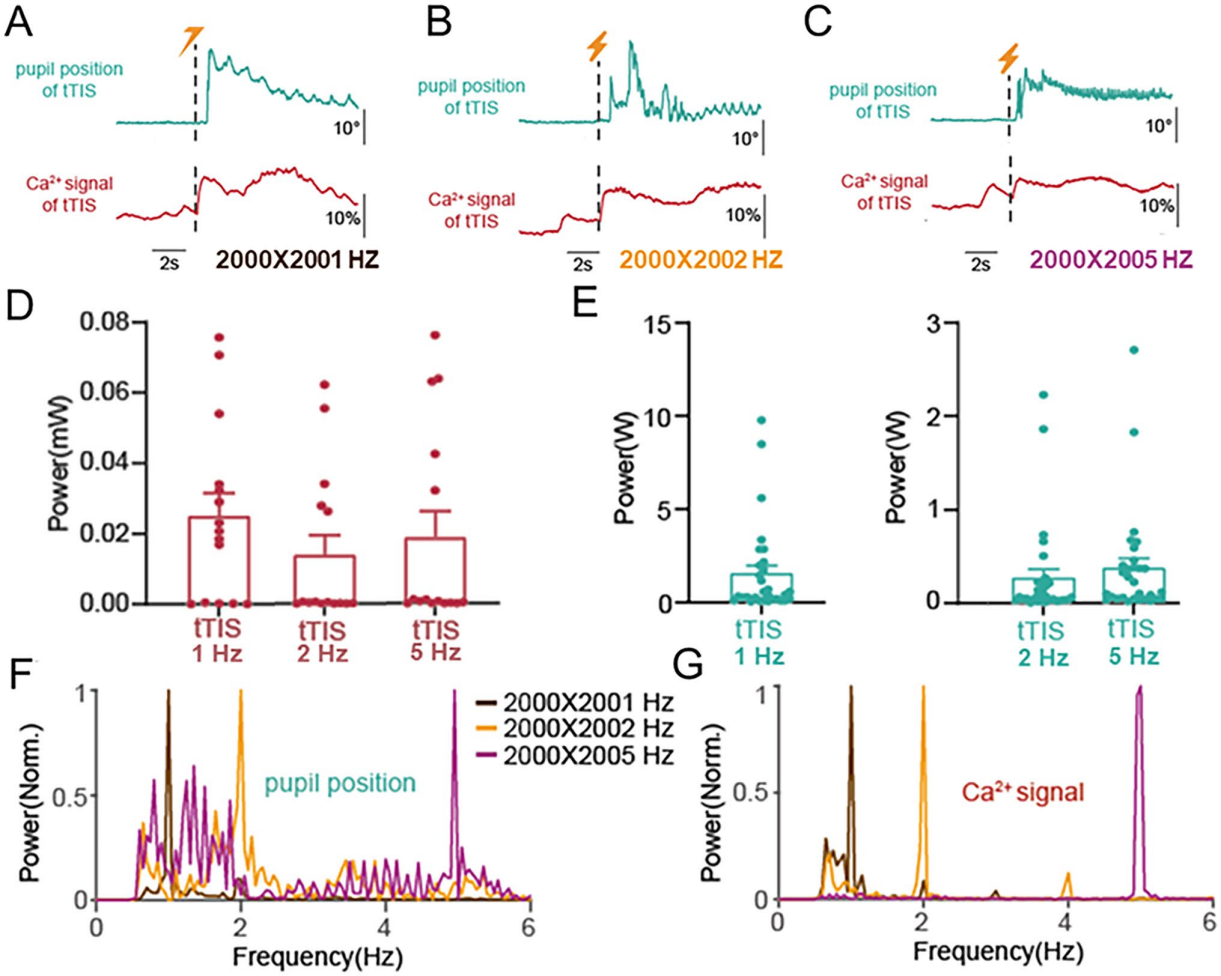
The relationship between stimulation, eye movements, and Ca^2+^ signals. **A–C** Example showing individual trials of eye movements and Ca^2+^ signals in the SC from one mouse. **D** Power spectrum of eye movement amplitudes in the 1-Hz band during stimulations. *n =* 6 mice, 30 trials per frequency difference. **E** Power spectrum of Ca^2+^ signals in the SC in the 1-Hz band during stimulations. *n =* 3 mice, 15 trials per frequency difference. **F** Normalized power spectrum of eye movement amplitudes during tTIS. *n =* 6 mice, 30 trials. **G** Normalized power spectrum of Ca^2+^ signals in the SC during tTIS. *n =* 3 mice, 15 trials.

Subsequently, we investigated whether eye movements and neural activity in the mouse SC can be modulated by manipulating the beat frequency of tTIS. tTIS was applied with 1 mA currents and 2 kHz carrier frequency, with beat frequencies of 1 Hz, 2 Hz, and 5 Hz, respectively (Fig. 6A–E). Our results suggested that the tTIS beat frequency can modulate periodic eye movements (Fig. 6F) by entraining the frequency of SC neural activity (Fig. 6G). These results indicate that the frequency of neural activity in the SC and eye movements can be regulated by tTIS beat frequency.

## Discussion

In this study, we investigated the effects of noninvasive tTIS regulating eye movement behaviors in mice. The tTIS was targeted at the SC, a subcortical region known to be involved in eye movement control [23]. Phantom experiments and *in silico* experiments were conducted to validate the tTIS effects. In addition, in mouse experiments, we implemented real-time monitoring of neural activity and eye movements during the stimulation phase. This comprehensive approach allowed us to closely study the effects of tTIS on eye movements, providing novel insights into the potential of this technique for targeted neural modulation.

Compared to tTIS, the traditional tACS activates a broader range of areas, including unintended regions. According to the literature [16], tTIS has been shown to regulate the movement frequency of the contralateral forepaw through stimulation of the motor area. Unlike the focus of their study on the direct relationship between the motor area and movement, our study investigated the neural activity in the SC, a much deeper brain region than the motor area, and the frequency of eye movements in response to tTIS. Our study provided evidence that tTIS over the SC can regulate neural activity and eye movements.

We constructed a phantom platform equipped with a specialized transcranial current stimulator to verify the capability of stimulating deep regions using tTIS. The electrical field effects of the tTIS current ratio and electrode distribution from the phantom model (Fig. 4) were in line with previous literature [12, 34]. The target was tuned by changing the current ratio without altering the electrode position. Furthermore, we examined the influence of electrode size using the phantom. We found that the size of tTIS electrodes had a lesser impact on the distribution of the simulation results (Fig. 3C *vs* Fig. 3E), in comparison with electrode position (Fig. 3A, C, G) and tTIS current ratio (Fig. 3I, G, K). Interestingly, the FEM simulation results showed that the electrical field in the mouse brain was significantly greater than that in the human brain reported in the literature [28]. Our results suggested that tTIS activated a more focal area than tACS when targeting deep brain regions (Fig. 3).

Our results from animal experiments showed that high-frequency currents without frequency difference cannot effectively stimulate the SC. With a 1 Hz beat frequency, the tTIS current had to exceed 0.8 mA to significantly enhance neural activity in the SC (Fig. 5). We found that the beat frequency of tTIS regulated neural activity, as well as the frequency of saccades. Specifically, in the low-frequency envelope range (<5 Hz), the neural activity and saccade frequencies were entrained by the tTIS beat frequencies (Fig. 6).

Our findings indicated the neural response (i.e., SC neural activity) and the behavioral output (i.e., saccade) exhibit a threshold effect (Fig. 5). Previous studies reported that when the SC is stimulated by a microcurrent, the saccade amplitude exhibits an “all or nothing” behavior [23], which means only when the current intensity reaches a certain threshold does the saccade amplitude start to increase linearly with the stimulation current intensity [23]. In our study, when the tTIS current was increased from 0.6 mA to 0.8 mA, a significant increase in both saccade amplitude and SC neuronal activity was recorded (Fig. 5). Surprisingly, when we further increased the current intensity, from 1.0 mA to 1.2 mA, the amplitude of the saccade kept increasing, rather than exhibiting an “all or none” behavior. This contradiction could be due to the differences in the number and range of neurons activated by tTIS compared to the invasive microcurrent stimulation in previous studies [16, 23]. We found a difference in threshold currents for Ca^2+^ imaging (0.8 mA) and eye movement (0.4 mA), which likely stems from the non-specific stimulation of neighboring brain regions. While tTIS can penetrate the skull and modulate deep brain activity, its precision in targeting small areas without affecting surrounding regions is currently limited. The fiber optic cable used for Ca^2+^ recordings was inserted into the middle and deep layers of the superior colliculus, capturing signals from a limited area. Since other regions within the SC also contribute to eye movements, non-specific stimulation by tTIS may explain this discrepancy. In the future, it is important to record neural activity over a broader spatial and frequency range in order to quantify the activity modes of neurons activated under different stimulation modes, such as tACS, tTIS, and invasive stimulation.

In this study, we utilized mice to investigate the spatial precision of tTIS. However, extrapolating these findings to human applications requires careful consideration. Firstly, we inevitably caused some skull damage while affixing the needle electrodes, and such skull defects can significantly affect the magnitudes of intracerebral electrical fields [42]. Secondly, there is a substantial size difference between the human and mouse brains, which leads to notable differences in field strength. Fortunately, recent studies have begun to explore the effects of tTIS in humans through both computational simulations [20] and human experiments [34, 43]. Notably, our comparison of tDCS and tTIS (Supplementary, Fig. 2) shows that tTIS can stimulate specific areas with greater precision and reduced electrical diffusion. This finding aligns with existing human studies and our results in mice, suggesting that tTIS has the potential for targeted activation and precise neuromodulation.

In the practical applications of tTIS, one fundamental question is whether tTIS can activate deep brain regions. Using our tTIS stimulator, we demonstrated that tTIS is capable of regulating the neural activity in the SC and generating saccade behaviors in mice (Figs 5 and 6). Our results showed that although tTIS with small currents was not sufficient to activate SC neural activity, tTIS can reliably activate such activity when the current intensity of tTIS exceeds 0.8 mA (Fig. 5). In tTIS practice on animals and humans, we have to particularly pay attention to the stimulation current intensity, due to safety considerations and the tolerance to current. For instance, for human experiments, tES intensity is not allowed to exceed 2 mA [44, 45]. Moreover, the layout of tTIS electrodes relies on the head shape of animals and humans. Designing an optimal tTIS strategy requires taking into consideration the stimulation objectives (e.g., depth of the brain region, the safety constraints, the desired intensity, and focality), the electrode montage (e.g., number, size, position), and the stimulation setup (e.g., the current injected to each electrode) [14]. With these considerations of optimal tTIS design, tTIS can be a promising tool for non-invasive stimulation of deep brain regions and has great potential in clinical applications.

## Conclusions

In conclusion, our study demonstrates that tTIS can modulate neural activity and eye movement behavior by targeting deep brain regions, such as the superior colliculus, with greater accuracy than conventional methods. Both computational modeling and *in vivo* experiments show that tTIS delivers more focused stimulation by adjusting beat frequency and current intensity, enabling specific modulation of neural activity and behavior. These findings underscore the significant potential of tTIS as a non-invasive neuromodulation tool, particularly for research and applications requiring precise targeting of deep brain regions.

## Supplementary Information

Below is the link to the electronic supplementary material.

Supplementary file1 (PDF 2125 kb)

## Data Availability

The datasets generated during and/or analyzed during the current study are available from the corresponding author upon reasonable request.
