# Supplementary material for: Transcranial temporal interference stimulation precisely targets deep brain regions to regulate eye movements
Source: Neurosci Bull. 2025 Apr 11;41(8):1390–402. doi: 10.1007/s12264-025-01387-3 (PMC12314165; doi:10.1007/s12264-025-01387-3)
Supplement: Supplementary file 1 — Supplementary file1 (PDF 2125 kb) [file 12264_2025_1387_MOESM1_ESM.pdf]

Supplementary

1. Design of the tTIS stimulator

The tTIS stimulator consists of two constant-current sources. Each constant current source consists of a direct digital synthesis signal generator, a group of constant-current feedback circuits, an inverse reference circuit, and other control circuits (Fig. S1A). The constant current feedback and inverse reference circuits could maintain a constant current output when connected to different loads. The alternating current in the equipment was generated by an inverse current drive. In channel 1 (CH1) (Fig. S1A), the signal generator generates a voltage waveform V_1 at a frequency of f1. After being amplified by an operational amplifier, it is output to a voltage-controlled current source (J1) to generate a current of the same waveform. The ratio of the magnitude of the current to the voltage of the signal generator is 2 mA/V. The same waveform is also applied to the second voltage-controlled current source (J2), and the generated current waveform is inverse to the waveform of J1. Similar to CH1, in channel 2 (CH2), the voltage-controlled current source (J3) generates the in-phase current waveform with the same waveform as the signal generator and the frequency is f2, and J4 generates the reverse current waveform. Here, a 1:1 output transformer is used as a means of isolation between channels. Based on the output characteristics of the transformer, the current applied by the constant current source to the transformer will be "copied" to the load.

We test the performance of tTIS by adjusting the Load ranging from 1–20 $k\Omega$ (Fig. S1B). The voltage waveform V_1 of CH1 was set to 2 kHz, resulting in a current I_1 between nodes a and b at the same frequency. The voltage waveform V_2 of CH2 was set to 2.001 kHz, resulting in a current I_2 between nodes a and b at the same frequency. The resistance bridge load consists of seven resistors with the same resistance value. An oscilloscope was used to measure the current amplitude between nodes a and b (Fig. S1C).

Each current source reliably drives constant current with different loads (Fig. S1D). The CH1 output is set to a sine wave at 2 kHz, 1.25 V, and a current at 2 kHz and 1 mA is generated between nodes a and b. The CH2 output is set to a sine wave at 2.001 kHz, 1.25 V, and a current at 2.001 KHz and 1 mA is generated between nodes a and b. The resistance bridge is composed of resistors with the resistance value of 2–40 $k\Omega$. At load resistances higher than 20 $k\Omega$, the current source eventually saturates. Fig. S1E indicates the Characterization of the output current for different set frequencies. The voltage waveform V_1 of CH1 was set to a sine wave at 2 kHz, resulting in a current between the a and b nodes of the same frequencies and with amplitudes that ranged between 100 μA and 5 mA. The voltage waveform V_2 of CH2 was set to a range of

frequencies between 2000.1 Hz and 2100 Hz, resulting in a current between a and b nodes of the same frequencies and with amplitudes that ranged between 100 μA and 5 mA. The resistance bridge is composed of resistors with a resistance of 1 $k\Omega$. (Note that the line plots of frequencies between 0.1 Hz and 10 kHz overlap).

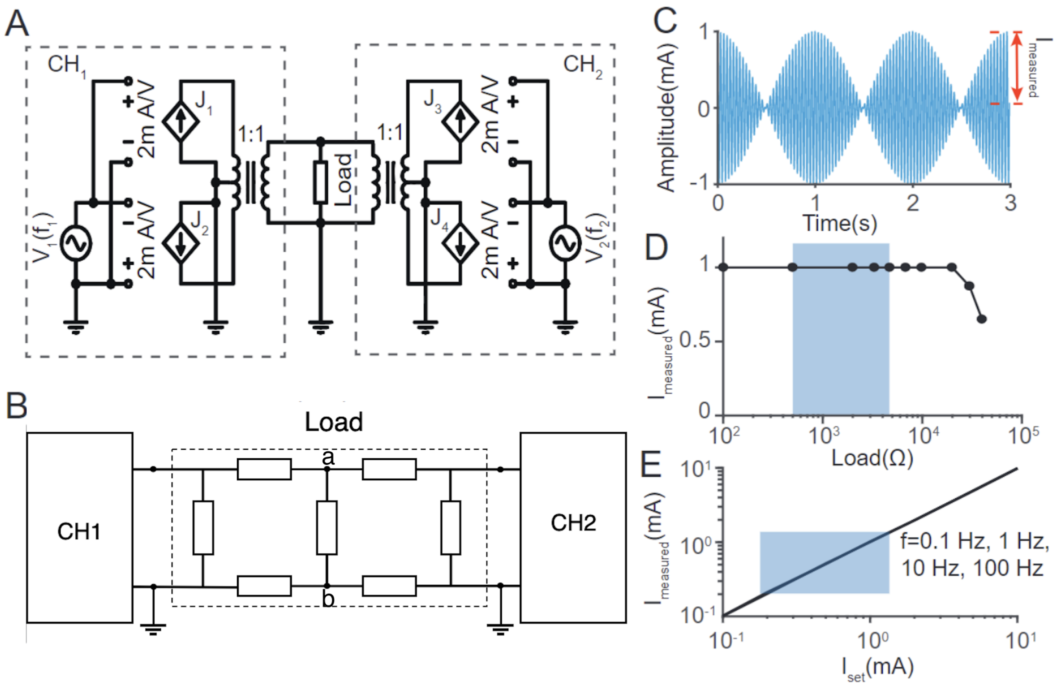

Figure S1. Design, implementation, and performance validation of tTIS stimulator. (A) Design of tTIS stimulator. (B) Schematic of the experimental setup to test the temporal interference stimulator by adjusting the Load. (C) The waveform of output current measured by an oscilloscope. (D) The output current intensity as a function of the impedance of load resistor. (E) The output current intensity as a function of set current intensity at different tTIS frequencies.

2. Human tES simulation

We utilized SimNIBS, a versatile software tool for simulating electric fields in the human brain, to construct a realistic head model based on the MNI152 template and the EEG 10-10 electrode system. The MNI152 template provides an anatomically standardized brain model, facilitating accurate spatial registration across different subjects, which makes it a widely accepted reference in neuroimaging studies. The EEG 10-10 system, an internationally recognized standard for electrode placement, was employed to define the positions of the stimulation electrodes. We used the CHARM function, a built-in feature of SimNIBS, to generate a Finite Element Method (FEM) model with default conductivity values for various tissues. The conductivities were assigned as follows: *0.126* S/m (white matter), *0.276* S/m (gray matter), *1.65* S/m (cerebrospinal fluid (CSF)), *0.01* S/m (skull), *0.465* S/m (scalp), *0.5* S/m (eye), *2.5* $e^{-14} S/m$ (air cavities), *1.0* S/m (saline). Electrode placements were set in the SimNIBS graphical user interface

(GUI), with two pairs of electrodes positioned at AF4-AF4 and F2-F1, each delivering 1 mA of current. We then calculated the electric field distribution using the relevant formulas [1–3] and visualized the results for further analysis. From the results (Fig.S2 B, C), we observe that tTIS generates a high-intensity electric field concentrated at the center of the electrode pairs, whereas the electric field produced by tDCS is more diffuse. The difference between tDCS and tTIS (Fig.S2 D) shows that tTIS can produce an electric field of equal intensity at deep target areas while activating fewer non-target areas.

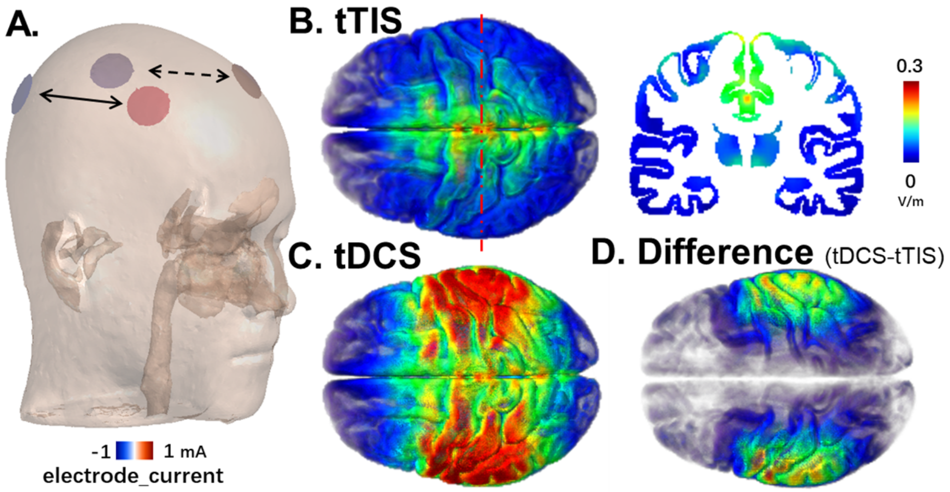

Figure S2. Visualization of tES simulation in human. (A) Simulation model based on the MNI152 template, displaying two electrode pairs: FC3-P3 and FC4-P4, each delivering a current of 1 mA. (B) Superior view of the electric field distribution in gray matter induced by tTIS, with a coronal view indicated by a red dotted line. (C) Superior view of the electric field distribution in gray matter induced by tDCS. (D) Superior view of the difference in electric field distribution between tDCS and tTIS.

References

[1] Grossman N, Bono D, Dedic N, Kodandaramaiah SB, Rudenko A, Suk HJ, et al. Noninvasive deep brain stimulation via temporally interfering electric fields. cell. 2017;169(6):1029-41.

[2] Thielscher A, Antunes A, Saturnino GB. Field modeling for transcranial magnetic stimulation: A useful tool to understand the physiological effects of TMS? 2015 37th Annual International Conference of the IEEE Engineering in Medicine and Biology Society (EMBC). 2015:222-5. Available from: `https://api.semanticscholar.org/CorpusID:13645270`.

[3] Wang M, Lou K, Liu Z, Wei P, Liu Q. Multi-objective optimization via evolutionary algorithm (MOVEA) for high-definition transcranial electrical stimulation of the human brain. NeuroImage. 2023;280:120331.
